# Digenic Inheritance and Gene-Environment Interaction in a Patient With Hypertriglyceridemia and Acute Pancreatitis

**DOI:** 10.3389/fgene.2021.640859

**Published:** 2021-04-16

**Authors:** Qi Yang, Na Pu, Xiao-Yao Li, Xiao-Lei Shi, Wei-Wei Chen, Guo-Fu Zhang, Yue-Peng Hu, Jing Zhou, Fa-Xi Chen, Bai-Qiang Li, Zhi-Hui Tong, Claude Férec, David N. Cooper, Jian-Min Chen, Wei-Qin Li

**Affiliations:** ^1^Department of Critical Care Medicine, Research Institute of General Surgery, Jinling Hospital, Medical School of Nanjing University, Nanjing, China; ^2^Department of Intensive Care Unit, The Affiliated Drum Tower Hospital, Medical School of Nanjing University, Nanjing, China; ^3^Department of Gastroenterology, Clinical Medical College, Yangzhou University, Yangzhou, China; ^4^Univ Brest, INSERM, EFS, UMR 1078, GGB, Brest, France; ^5^Service de Génétique Médicale et de Biologie de la Reproduction, CHRU Brest, Brest, France; ^6^School of Medicine, Institute of Medical Genetics, Cardiff University, Cardiff, United Kingdom

**Keywords:** acute pancreatitis, alcohol-induced hypertriglyceridemia, *LPL* gene, *APOA5* gene, gene-environment interaction

## Abstract

The etiology of hypertriglyceridemia (HTG) and acute pancreatitis (AP) is complex. Herein, we dissected the underlying etiology in a patient with HTG and AP. The patient had a 20-year history of heavy alcohol consumption and an 8-year history of mild HTG. He was hospitalized for alcohol-triggered AP, with a plasma triglyceride (TG) level up to 21.4 mmol/L. A temporary rise in post-heparin LPL concentration (1.5–2.5 times of controls) was noted during the early days of AP whilst LPL activity was consistently low (50∼70% of controls). His TG level rapidly decreased to normal in response to treatment, and remained normal to borderline high during a ∼3-year follow-up period during which he had abstained completely from alcohol. Sequencing of the five primary HTG genes (i.e., *LPL*, *APOC2*, *APOA5*, *GPIHBP1* and *LMF1*) identified two heterozygous variants. One was the common *APOA5* c.553G > T (p.Gly185Cys) variant, which has been previously associated with altered TG levels as well as HTG-induced acute pancreatitis (HTG-AP). The other was a rare variant in the *LPL* gene, c.756T > G (p.Ile252Met), which was predicted to be likely pathogenic and found experimentally to cause a 40% loss of LPL activity without affecting either protein synthesis or secretion. We provide evidence that both a gene-gene interaction (between the common *APOA5* variant and the rare *LPL* variant) and a gene-environment interaction (between alcohol and digenic inheritance) might have contributed to the development of mild HTG and alcohol-triggered AP in the patient, thereby improving our understanding of the complex etiology of HTG and HTG-AP.

## Introduction

Acute pancreatitis (AP) is a common critical disease, has poor outcomes and a high mortality rate, and requires complex clinical management (Hazra and Gulliford, 2014; Lee and Papachristou, 2019). The etiology of AP is diverse (AGA, 2007; IAP/APA, 2013). In Western countries, gallstones and alcohol consumption represent the most common causes (Sekimoto et al., 2006; Yadav and Lowenfels, 2013) whereas in China, hypertriglyceridemia (HTG) rather than alcohol consumption is the second leading cause (Yin et al., 2015; Jin et al., 2019). Hypertriglyceridemia-induced acute pancreatitis (HTG-AP) has been defined as pancreatitis with a triglyceride (TG) level of up to 1000 mg/dL (11.3 mmol/L) alone, or 500 mg/dL (5.65 mmol/L) accompanied by lipemic or lactescent (milky in appearance) blood, after excluding other etiologies (Zafrir et al., 2018). By comparison with biliary acute pancreatitis, HTG-AP occurs earlier, is more severe, has a higher recurrence rate, and affects more males than females (Yin et al., 2015; Li et al., 2018; Jin et al., 2019).

HTG can be broadly divided into primary and secondary categories in terms of etiology (Yuan et al., 2007; Rygiel, 2018). Secondary HTG is primarily related to lifestyle factors such as heavy alcohol consumption and smoking, physiological factors such as pregnancy, or comorbid pathological conditions such as obesity, diabetes mellitus, hypothyroidism, and renal dysfunction (Black and Sprecher, 1993; Merkel et al., 2002). By contrast, primary HTG has been found to be mainly due to genetic defects in five lipid metabolism-related genes (Nawawi et al., 2020) namely *LPL* (lipoprotein lipase, OMIM #609708), *LMF1* (lipase maturation factor 1, OMIM #611761), *GPIHBP1* (glycosylphosphatidylinositol-anchored high density lipoprotein-binding protein 1, OMIM #612757), *APOA5* (apolipoprotein A-V, OMIM #606368), and *APOC2* (apolipoprotein C-II, OMIM #608083). LPL is the key enzyme catabolizing triglyceride (Olivecrona, 2016). LMF1 functions in the maturation of LPL (Peterfy et al., 2007). GPIHBP1 mediates the transmembrane transport and binding of LPL (Beigneux et al., 2007). APOA5 and APOC2 act as basic activators of LPL activity (Breckenridge et al., 1978; Pennacchio et al., 2001).

The abovementioned dichotomous classification of HTG provides a useful framework for understanding the etiology of HTG. However, in most cases, the etiology of HTG is complex, and often involves primary-primary (i.e., gene-gene) and primary-secondary (i.e., gene-environment) interactions (Wang et al., 2008; Hegele et al., 2014; Cole et al., 2015; Serveaux Dancer et al., 2018; Chen et al., 2019; Dron et al., 2019; Pu et al., 2020). Herein, we describe the clinical, genetic and functional analysis of a patient with alcohol-triggered HTG-AP, which serves to provide us with several novel insights into the complex etiology of HTG.

## Materials and Methods

### Ethics Statement

This study was approved by the Ethics Committee of Jinling Hospital. Informed consent for publication was obtained from all participants.

### Patient

The male Chinese patient experienced sudden upper abdominal pain after consuming ∼150 g alcohol on October 29, 2017. He was initially admitted to a local hospital: physical examination revealed epigastric tenderness, without Gray Turner’s or Cullen’s sign; amylase increased from 305 U/L to 1,294 U/L over one night; a computed tomography (CT) scan indicated AP. He was transferred to our severe acute pancreatitis treatment center in Jinling Hospital on October 31, 2017. Diagnosis of AP and classification of the disease in terms of clinical severity were made in accordance with the modified Atlanta classification (Banks et al., 2013).

### Analysis of Plasma Lipid Profiles

Fasting blood samples were collected for this analysis. Plasma lipid profiles including TG, total cholesterol (TC), high-density lipoprotein cholesterol (HDL-C) and low-density lipoprotein cholesterol (LDL-C), as well as plasma glucose were measured enzymatically on an automatic analyzer (Hitachi High-Tech, 7600–120, Japan).

### Analysis of LPL Mass and Activity in Post-heparin Plasma

Blood samples were collected after overnight fasting and 10 min after intravenous heparin injection (60 IU/kg body weight). LPL mass was determined using a human LPL Elisa kit (TSZ Biological Trade Co., Ltd., San Francisco, CA, United States) in accordance with the manufacturer’s instructions. To measure LPL activity, plasma total lipase activity and hepatic lipase activity were first determined by a LPL-mediated lipolysis reaction; this was performed by means of a free fatty acid (FFA) release assay kit [Wako kit# NEFA-HR(2), Japan], using TG-rich plasma from *GPIHBP1*-deficient (*GPIHBP1^–/–^*) mice as the lipolytic substrate (Li et al., 2020; Shi et al., 2020). It should be noted that, for the hepatic lipase activity assay, the sample was pretreated with 1 M NaCl and incubated for 60 min at 4°C in order to inactivate LPL. Subtraction of the hepatic lipase activity from the total lipase activity then yielded a measure of the plasma LPL activity. All assays were performed in five replicates. Eight healthy volunteers from our center (four males and four females with a mean age of 28 years) were used as controls.

### Sanger Sequencing

Genomic DNA was extracted from a blood sample using the TIANamp Blood DNA kit (TIANGEN Biotech, Beijing, China) according to the manufacturer’s instructions. All coding and proximal intronic regions of the *LPL*, *APOC2*, *APOA5*, *GPIHBP1*, and *LMF1* genes were analyzed by Sanger sequencing as previously described (Chen et al., 2019; Pu et al., 2020). All primer sequences are available upon email request.

### Reference Sequences, Variant Nomenclature, Public Databases, and Pathogenicity Prediction for Missense Variants

NM_000237, NM_000483, NM_001371904, NM_178172, and NM_022773 were used as the mRNA reference sequences for the *LPL*, *APOC2*, *APOA5*, *GPIHBP1*, and *LMF1* genes, respectively. Variant nomenclature followed the Human Genome Variation Society (HGVS) recommendations (den Dunnen et al., 2016). Variant population allele frequencies were in accordance with the Genome Aggregation Database (gnomAD)^1^ (Karczewski et al., 2020). The Human Gene Mutation Database (HGMD)^2^ (Stenson et al., 2020), ClinVar^3^, the *LPL* locus-specific mutation database^4^ and PubMed^5^ were used to establish whether or not a given variant had been previously described. LPL amino acid sequences from diverse vertebrate species were taken from https://www.ncbi.nlm.nih.gov/home/proteins/to evaluate the evolutionary conservation status of the p.Ile252 position. As previously described (Guéguen et al., 2020), we used the PP3 rule established by VarSome (Kopanos et al., 2019) to predict the pathogenicity of missense variants; the PP3 verdict was based upon computational evidence derived from 13 *in silico* algorithms (i.e., BayesDel_addAF, DANN, DEOGEN2, EIGEN, FATHMM-MKL, M-CAP, MutationAssessor, MutationTaster, MVP, PrimateAI, REVEL, and SIFT).

### Plasmid Construction, Cell Culture, and Transfection

The wild-type *LPL* cDNA expression vector has been previously described (Li et al., 2020; Shi et al., 2020) and was used here to generate the mutant [c.756T > G (p.Ile252Met)] expression vector by site-directed mutagenesis. The mutated site was verified by Sanger sequencing. Human embryonic kidney 293T (HEK293T) cells (ATCC, CRL-3216) were cultured in Dulbecco’s Modified Eagle’s Medium (DMEM, Lonza, C11995500BT) with 10% Fetal Bovine serum (FBS) and 1% penicillin-streptomycin at 37°C with 5% CO_2_. Transfections were performed using Lipofectamine 3000 (Thermo, L3000015) in 6-well plates, essentially as previously described (Yu et al., 2006; Han et al., 2020). It should, however, be noted that in addition to the wild-type expression vector, mutant expression vector and empty vector transfection groups, a new group mimicking the heterozygous state of the variant was included here. More specifically, whereas the former three groups each used 3 μg of the respective plasmid for transfection, the new group used a mix of 1.5 μg wild-type plasmid and 1.5 μg mutant plasmid for transfection. Moreover, whereas the transfection time (i.e., from addition of the plasmid into the culture media to the change to fresh media) was invariably maintained for 6 h, downstream treatments were different in accordance with the subsequent Western blot analysis. Thus, for analysis of LPL expressed in the transfected cells without heparin treatment, the cells were cultured for 48 h with the fresh media before cell lysate preparation. For analysis of LPL expressed in the transfected cells and secreted into the media, the fresh medium was further changed to a 500 μL heparin-DMEM mixture (the ratio of DMEM and heparin was 500:8, whereas heparin was 20 units/mL) per well; the cells were cultured for an additional 30 min. Three independent transfections were performed for each experiment.

### Western Blot

Transfected cell medium was centrifuged at 12,000 r/min for 5 min to remove cells and cell debris and stored at –80°C for later analysis. Transfected cells were treated with 80 μL RIPA Lysis Buffer (Beyotime, P0013B, China) and 8 μL protease inhibitor (PI, Roche, 4693116001) and centrifuged at 12,000 r/min for 5 min; the supernatant was stored at −80°C for subsequent analysis. Protein concentration was determined by BCA kit.

30 μg cell lysates or 200 μL cell media were mixed with SDS-PAGE Protein Loading Buffer (reducing) and incubated at 95°C for 10 min. It should be noted that the cell media were firstly concentrated in the upper layer of the polyacrylamide gel (concentration of 12%) by repeated sample loading (40 μL × 5 times; each time run at 90 V for 15 min). Proteins were separated by SDS-PAGE (10% acrylamide gel, 130 V, 90 min), transferred onto a nitrocellulose membrane (220 mA, 120 min), and then blocked with 5% BSA (room temperature, 60 min). After washing with 0.2% Tris-Buffered Saline with Tween 20 (TBST), membranes were incubated overnight with mouse anti-LPL antibody (Santa, sc-73646; 1:200 dilution) at 4°C. After washing with 0.2% TBST, membranes were incubated for 1 h with goat anti-mouse (Abcam, ab6728; 1:2000 dilution) IgG H&L (HRP) antibody, and again washed three times. After 5 min incubation with chemiluminescent HRP substrate (Thermo Fisher Scientific), bands were visualized and analyzed by the Chemidoc XRS System, Image Lab Software (Clinx Science Instruments, Shanghai, China). The protein bands were quantified by densitometry and normalized to GAPDH (Santa, sc-69778; 1:2000 dilution).

### LPL Activity Analysis in Transfected Cell Medium and Lysates

This was performed in the same way as for the analysis of LPL activity in post-heparin plasma, but without the treatment with 1M NaCl, as there was no hepatic lipase activity in the cell medium or lysates. Each assay was performed in five replicates.

### Statistical Analysis

Results from Western blot and LPL activity analysis in transfected cell medium and lysates are shown as mean ± standard deviation (SD) from at least three independent experiments. The differences between groups were evaluated by means of a Student’s *t*-test using SPSS 24.0, with *p*-values less than 0.05 being considered indicative of statistical significance.

## Results

### Clinical Findings in the Patient

**FIGURE 1:**
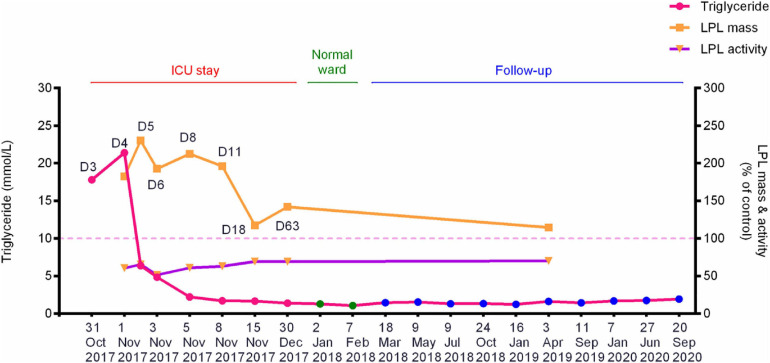
Dynamic changes of the fasting plasma TG levels, post-heparin LPL mass and LPL activity during hospitalization and follow-up periods of the patient with alcohol-triggered HTG-AP. Day 3 (D3), numbered by reference to the time of disease onset, corresponds to the date when the patient was transferred to our service. See Table 1 for the precise values of the three parameters. HTG-AP, hypertriglyceridemia-induced acute pancreatitis; LPL, lipoprotein lipase; ICU, intensive care unit.

**FIGURE 2:**
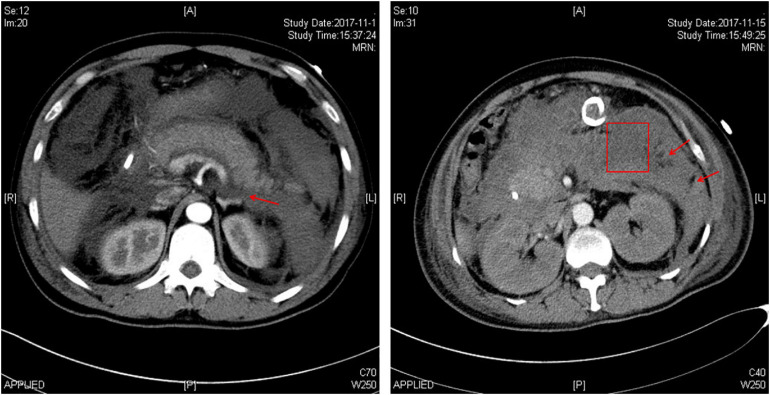
Abdominal computed tomography images of the patient showing the affected pancreas. The left image was taken on the third day of onset of acute pancreatitis, showing peripancreatic exudation (arrow) and the enlarged pancreas with adjacent water density shadow. The right image was taken on the eighteenth day of disease onset, showing the occurrence of infected pancreatic necrosis. The box indicates a partial necrotic pancreas whilst the arrows indicate infective necrosis.

The patient suffered from an attack of AP that appears to have been triggered by the consumption of ∼150 g alcohol on October 29, 2017. On day 3 (October 31, 2017) of the disease onset, he was transferred from the local hospital to our therapy center at Jinling Hospital, one of the biggest referral centers for severe AP treatment in China. In our center, the patient was more precisely diagnosed as having HTG-AP on the basis that his TG level was as high as 17.8 mmol/L (Figure 1). Figure 2 shows computed tomography images of the affected pancreas from two time points during the early days of the disease. Continuous renal replacement therapy (CRRT) and pulmonary ventilation were performed to rescue renal and respiratory function, respectively. After a 64-day hospitalization in our intensive care unit, and 40 days on a normal ward, he was discharged on February 11, 2018.

**TABLE 1:** Plasma lipid profiles and post-heparin LPL mass and activity in the patient.

**Date of measurement**	**Time point**	**Glucose (mmol/L)**	**TG (mmol/L)**	**TC (mmol/L)**	**HDL-C (mmol/L)**	**LDL-C (mmol/L)**	**LPL mass (% of normal)^†^**	**LPL activity (% of normal)^‡^**
31 October 2017	Admission (D3)^§^	9.3	17.80	13.69	2.87	9.21	ND	ND
1 November 2017	D4	9.3	21.40	11.30	1.79	8.49	339.8 (182.3)	1.0445 (60.6)
2 November 2017	D5	10.2	6.39	5.32	1.27	3.09	429.0 (230.1)	1.1287 (65.5)
3 November 2017	D6	9.4	4.87	3.98	0.92	2.37	358.9 (192.5)	0.8841 (51.3)
5 November 2017	D8	9.1	2.23	3.34	0.38	1.43	395.6 (212.2)	1.0520 (61.0)
8 November 2017	D11	10.6	1.83	2.67	0.23	1.15	365.5 (196.1)	1.0881 (63.1)
15 November 2017	D18	8.1	1.93	2.47	0.26	1.71	219.2 (117.6)	1.1965 (69.4)
30 December 2017	D63	6.6	1.40	3.00	0.46	1.78	264.6 (141.9)	1.1939 (69.2)
2 January 2018	Switch to normal ward	6.6	1.30	3.49	0.90	2.30	ND	ND
7 February 2018	Discharge	5.6	1.08	4.58	1.10	3.00	ND	ND
18 March 2018	Follow-up	6.07	1.46	5.51	1.2	3.19	ND	ND
9 May 2018	Follow-up	4.95	1.55	5.22	1.23	3.28	ND	ND
9 July 2018	Follow-up	4.07	1.32	4.43	1.00	2.8	ND	ND
24 October 2018	Follow-up	4.32	1.35	3.8	1.13	2.23	ND	ND
16 January 2019	Follow-up	5.22	1.25	4.71	1.38	2.75	ND	ND
3 April 2019	Follow-up	4.93	1.63	5.45	1.52	3.13	213.9 (114.7)	1.2100 (70.2)
11 September 2019	Follow-up	4.64	1.44	4.95	1.33	2.84	ND	ND
7 January 2020	Follow-up	5.02	1.70	5.04	1.19	3.37	ND	ND
27 June 2020	Follow-up	4.70	1.76	5.31	1.44	3.14	ND	ND
20 September 2020	Follow-up	4.97	1.94	5.28	1.35	3.39	ND	ND

*^†^Normal value (derived from eight healthy controls) was 186.4 ± 56.1 U/L. ^‡^Normal value (derived from eight healthy controls) was 1.7242 ± 0.4358 mEq/L/h. ^§^Admission to our service was on day 3 of disease onset. D, day; ICU, intensive care unit; TG, triglyceride; TC, total cholesterol; HDL-C, high-density lipoprotein cholesterol; LDL-C, low-density lipoprotein cholesterol; ND, not detected.*

### Personal and Family History of the Patient

The patient had an 8-year history of mild HTG (a fasting plasma TG level of ∼5 mmol/L) and a 20-year history of continuous consumption of ∼100 g alcohol per day. He had no prior attacks of AP. He had had no cholelithiasis, diabetes or other diseases that are known to cause HTG. He also had no history of drug abuse. There was no family history of AP. His elder brother was, however, reported to have drunk >80 g alcohol per day for >20 years and had a borderline high TG (2.08 mmol/L).

### Long-Term Measurement of TG Levels and Post-heparin LPL Mass and Activity in the Patient

Plasma lipid profiles and post-heparin LPL mass and activity in the patient were determined regularly during his hospital stay and follow-up period (Table 1 and Figure 1). The highest LPL mass (429.0 U/L; ∼2.3 times higher than normal controls) was observed on day 5 whereas the lowest LPL activity (0.8841 mEq/L/h; ∼50% of normal controls) was observed on day 6 of AP onset. It should be noted that during the follow-up period, the patient abstained from both drinking and smoking as advised; his TG levels remained at normal or at most borderline high levels. The normal post-heparin LPL mass and activity values were averages derived from eight healthy controls (Table 1). Additionally, post-heparin LPL mass and activity in the elder brother (187.6 U/L and 1.7112 mEq/L/h, respectively) and son (274.3 U/L and 1.7338 mEq/L/h, respectively) were found to be within the normal range (184.4 ± 56.1 U/L and 1.7242 ± 0.4358 mEq/L/h, respectively).

### Genetic Findings in the Patient and His Family Members

**FIGURE 3:**
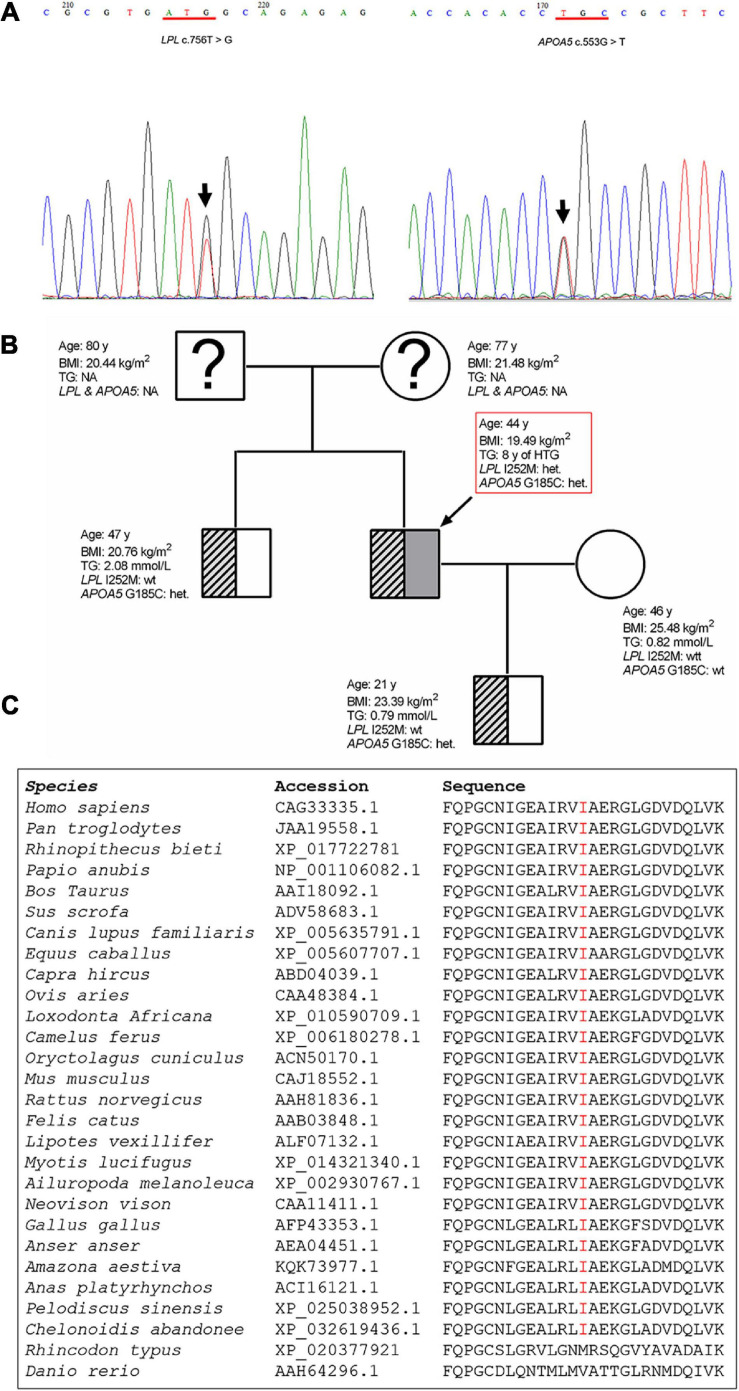
Genetic variants detected in the patient and the family tree. **(A)** Sanger sequencing electropherogram showing the novel *LPL* c.756T > G (p.Ile252Met) variant and the known common *APOA5* c.553G > T (p.Glu185Cys) variant identified in the patient. The variants are indicated by arrows. **(B)** Arrow denotes the patient. Genotype status with respect to the *APOA5* c.553G > T and *LPL* c.756T > G variants, age, BMI and TG levels are provided for each subject. **(C)** Alignment of partial LPL amino acid sequences spanning the p.252 site. *LPL*, lipoprotein lipase; *APOA5*, apolipoprotein A5; BMI, body mass index; het, heterozygous; wt, wild-type; TG, triglyceride; NA, not analyzed.

Sequencing of the *LPL*, *APOA5*, *APOC2*, *LMF1*, and *GPIHBP1* genes in the patient led to the identification of two heterozygous variants (Figure 3A). One was the common *APOA5* c.553G > T (p.Gly185Cys; rs2075291) variant, which has previously been associated with altered TG levels (Kao et al., 2003; Tang et al., 2006) as well as HTG-AP (Pu et al., 2020). The other is a rare variant in the *LPL* gene, c.756T > G (p.Ile252Met). This latter variant has an allele frequency of 0.000003978 (1/251358) in the gnomAD dataset (see text footnote 1). It does not, however, appear in the literature nor has it been registered in HGMD (see text footnote 2), ClinVar (see text footnote 3) or the *LPL* locus-specific mutation database (see text footnote 4) (as of 22 September 2020). The *LPL* p.Ile252 position is strictly conserved during mammalian and avian evolution (Figure 3C) and the p.Ile252 variant was predicted to be “pathogenic” in accordance with the PP3 rule established by VarSome (Kopanos et al., 2019). This pathogenicity assessment was based on nine independent predictions from BayesDel_addAF, DANN, DEOGEN2, FATHMM-MKL, M-CAP, MutationAssessor, MutationTaster, REVEL, and SIFT against 3 benign predictions from EIGEN, MVP, and PrimateAI.

The patient’s older brother, wife and son were also available for genetic analysis. The brother and son were found to carry the common *APOA5* p.Gly185Cys variant (Figure 3B).

### *In vitro* Functional Characterization of the *LPL* p.Ile252Met Missense Variant

**FIGURE 4:**
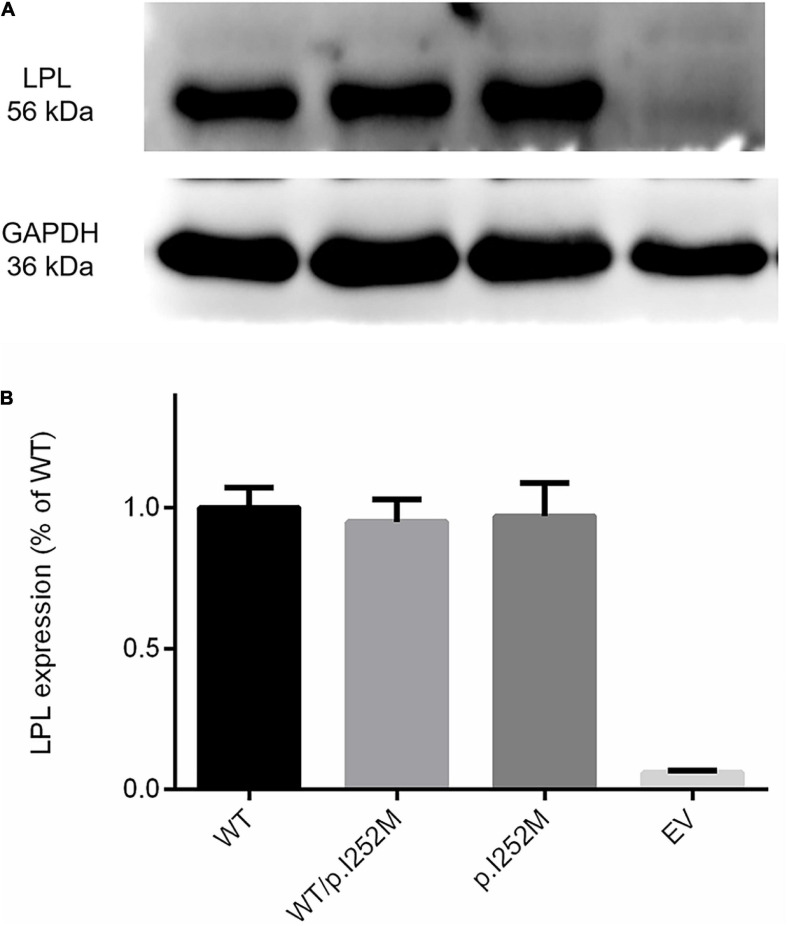
Western blot analysis of LPL expression in transfected HEK293T cells without heparin treatment. **(A)** Results were the average taken from three independent experiments. **(B)** LPL, lipoprotein lipase; WT, wild-type; EV, empty vector.

**FIGURE 5:**
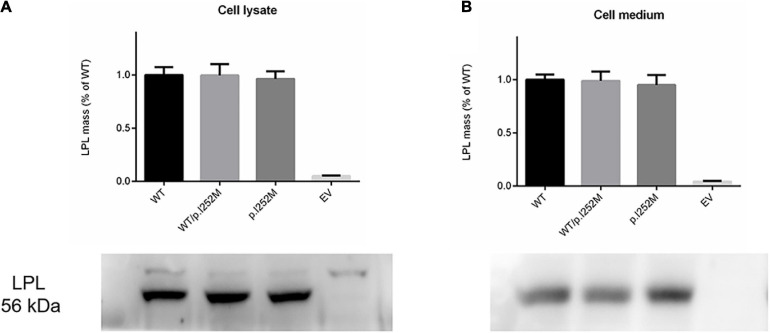
Western blot analysis of post-heparin LPL expression in cell lysates **(A)** and media **(B)** of transfected HEK293T cells. Results were the average taken from three independent experiments. LPL, lipoprotein lipase; WT, wild-type; EV, empty vector.

**FIGURE 6:**
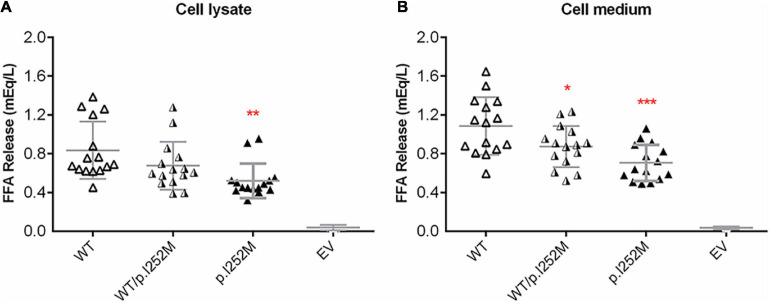
Post-heparin LPL activity in transfected HER293T cell lysates **(A)** and medium **(B)**. Results were expressed as mean ± SD from three independent transfections, with each transfection being performed in five replicates. LPL, lipoprotein lipase; WT, wild-type; EV, empty vector. *p < 0.05; **p < 0.01; ***p < 0.001.

We firstly performed Western blot analysis of LPL expressed in the transfected cells without heparin treatment. No difference was found between the wild-type, wild-type/mutant, and mutant expression vector groups (Figure 4). We then performed western blot analysis of LPL present in the transfected cell lysates and those secreted into the culture medium after heparin treatment (exogenous heparin promotes the release of cell surface-bound LPL into the medium). No difference was found between the three groups in either case (Figure 5). However, the mutant expression vector yielded reduced LPL activity in both cell lysates and medium. More specifically, the reduction from the “homozygous” mutant expression vector was approximately double that from the “heterozygous” mutant expression vector and, if expressed in the context of alleles, we estimate that the mutation caused a 40% loss of enzyme activity as compared to the wild-type (Figure 6). Taken together, the *LPL* p.Ile252Met variant served to reduce the enzymatic activity of the LPL protein but did not exert a dominant-negative effect.

## Discussion

In the present study, we report the detailed clinical, biochemical and family analysis of a patient with alcohol-triggered HTG-AP as well as the *in vitro* functional analysis of the rare *LPL* p.Ile252Met variant. We obtained long-term data on TG levels and post-heparin LPL mass and activity for the patient and performed *in vitro* functional analysis of the missense variant of interest in both the “heterozygous” and “homozygous” state and under different experimental conditions (with and without heparin treatment). Integration of these findings provides several novel insights into the complex etiology of HTG and HTG-AP.

First, the patient was found to carry a known common risk single nucleotide polymorphism (SNP) (i.e., *APOA5* c.553G > T (p.Gly185Cys); rs2075291) and a rare variant [i.e., *LPL* c.756T > G (p.Ile252Met)]. *APOA5* c.553G > T has an allele frequency of 0.06765 in East Asians (according to gnomAD); and in the Asian population, c.553T carriers displayed an increased risk of HTG as compared with c.553 GG carriers, with the overall random effects odds ratio (OR) being 3.55 [95% confidence interval (CI) 2.46 to 5.13] in the context of a dominant genetic model (He et al., 2016). By reference to the definitions employed by Manolio et al. (2009), the *APOA5* c.553G > T variant may be best described as a common genetic variant with moderate genetic effect (on HTG) in the Asian population. By contrast, the *LPL* c.756T > G (p.Ile252Met) variant is extremely rare in normal populations (allele frequency of 0.000003978 according to gnomAD) and has not previously been reported in HTG patients. The p. Ile252 site is conserved across the classes Mammalia and Aves (Figure 3C), suggesting its functional importance for protein structure and function. Consistent with the conservation of Ile252 over 300 million years of evolution, p.Ile252Met was predicted to be pathogenic by 9 of the 13 *in silico* algorithms employed by VarSome (Kopanos et al., 2019). Further, post-heparin LPL activity in the patient is approximately 70% of normal (Table 1 and Figure 1). It should be noted here that the post-heparin LPL activity in the patient was, in fact, a reflection of the net result of the complex interaction between the *LPL* gene and its regulating genes (such as *APOC2*, *APOA5*, *GPIHBP1*, and *LMF1*) as well as environmental and lifestyle factors. Thus, to decipher the direct effect of the p.Ile252Met variant on LPL protein and function, we further performed an *in vitro* transfection study, which demonstrated that the mutant p.Ile252Met allele caused a 40% loss of enzyme activity as compared to the wild-type allele (Figure 6). Based upon these observations, we believe that it is reasonable to define the *LPL* p.Ile252Met variant as a rare genetic variant that confers a rather moderate effect on LPL function (and presumably on HTG). Taken together, the patient was determined to have a digenic basis for his previous mild HTG and the current severe HTG-AP. To the best of our knowledge, this is the first time that *trans* heterozygosity for a common variant and a rare variant, both of which have a moderate effect on HTG, has been described in this disease. In this regard, it is pertinent to mention that in the context of TG-related genes, common variants are generally thought to have a small genetic effect whereas rare variants are thought to have a strong genetic effect (Johansen and Hegele, 2011; Dron and Hegele, 2020). Additionally, it should also be appreciated that the *APOA5* p.Gly185Cys variant appeared to amplify the effect of the *LPL* p.Ile252Met variant on reducing LPL activity, as the reduction of the plasma LPL activity in the patient (30% lower than normal) was 10% higher than the reduction of the LPL activity conferred by the “heterozygous” mutant *LPL* variant *in vitro* (20% lower than wild-type). Although care should always be exercised when comparing *in vivo* data directly with *in vitro* data, such a difference has a strong biological basis. The *APOA5* p.Gly185Cys impairs the function of APOA5; since APOA5 is an activator of LPL, this loss-of-function leads in turn to less activated LPL (Dorfmeister et al., 2008; Huang et al., 2012; Sharma et al., 2014; Chang et al., 2018).

Second, alcohol abuse is the most important environmental factor causing HTG owing to its impact on very-low-density lipoprotein secretion, lipolysis and free fatty acid fluxes from adipose tissue to the liver (Van de Wiel, 2012; Klop et al., 2013; Zemánková et al., 2015). Herein, having performed long-term follow-up of the TG levels in the patient, we were able to report a previously undescribed scenario: whereby the consumption of 150 g alcohol apparently triggered the attack of severe HTG-AP, the daily consumption of ∼100 g alcohol over 20 y is likely to have contributed to his pre-existing mild-HTG. This latter postulate was based on the observation that the TG levels in the patient remained normal, or at most borderline high, during the nearly 3-year follow-up period, during which the patient had completely abstained from drinking alcohol. Further follow-up studies are, however, needed to draw a firm conclusion.

Third, most of the HTG-associated rare *LPL* missense variants reported to date result in a complete or >90% functional loss of the affected allele, with this functional loss often being linked to upstream effects on protein synthesis, transport and secretion rather than to an isolated effect on enzyme activity (Rouis et al., 1996; Murano et al., 2005; Yu et al., 2006; Liu et al., 2016; Shi et al., 2020). To date, we are aware of only one rare *LPL* missense variant in the literature (i.e., a homozygous p.Ala203Thr variant found in a LPL-deficient patient) that specifically disrupted enzyme activity (Beg et al., 1990). However, both *in vivo* and *in vitro* findings (Beg et al., 1990) pointed to the p.Ala203Thr variant experiencing a complete loss of enzymatic activity. By contrast, the *LPL* p.Ile252Met mutant currently described here retained 60% enzyme activity as compared to its wild-type counterpart. Interestingly, p.Ile252Met occurred within a region of LPL, encompassing amino acid positions 243 to 266, that plays a crucial role in determining lipase substrate specificity (Dugi et al., 1995).

Lastly, we observed that whereas LPL mass was significantly increased in the patient during the acute phase of HTG-AP, LPL activity remained essentially unchanged at 70% normal (Figure 1). We surmised that this may be due to a dominant-negative effect of the mutant *LPL* p.Ile252Met allele, given that LPL is active mainly as a homodimer (Griffon et al., 2009; Arora et al., 2019; Beigneux et al., 2019). To explore this postulate, we mimicked the heterozygous state of the variant by mixing an equal amount of wild-type and mutant expression vectors in the transfection experiments. As shown in Figure 6, the impact of the “homozygous” variant was approximately double that of the “heterozygous” variant in terms of reducing LPL activity, thereby excluding such a postulate. It might also be that under stress of severe HTG, a positive feedback cycle stimulated LPL synthesis. However, a significant fraction of rapidly synthesized LPL proteins may not fold correctly and hence might not function normally. In this regard, it did not escape our attention that in the aforementioned LPL-deficient patient carrying the *LPL* p.Ala203Thr variant, higher than normal LPL mass concentrations were present in both pre- and post-heparin samples (Beg et al., 1990). It should, however, be noted that no information was available as to whether or not the patient had AP when the analysis was performed. Moreover, unlike our patient who had retained 70% normal LPL activity, the homozygous p.Ala203Thr carrier had no LPL activity at all. Consequently, we surmise that in this latter patient, the increased LPL mass may more likely be due to a feedback stimulus resulting from the absence of LPL activity. Irrespective of the underlying reasons, the increased LPL mass in both patients is consistent with the *in vitro* evidence that the respective *LPL* missense variants did not affect either protein synthesis or secretion.

The above notwithstanding, it should be emphasized that this study focused on a single case with HTG-AP. Further studies of more cases are required for us to obtain a better understanding of the genotype-phenotype relationships underlying this rare entity. Moreover, although loss-of-function *APOA5* variants have previously been shown to lead to less activated LPL by several studies (Dorfmeister et al., 2008; Huang et al., 2012; Sharma et al., 2014; Chang et al., 2018), co-transfection experiments of *APOA5* p.Gly185Cys and *LPL* p.Ile252Met expression vectors would be expected to yield confirmatory evidence for the gene-gene interaction between the two variants.

## Conclusion

We provide concrete evidence that gene-gene and gene-environment interactions are responsible for causing mild HTG and HTG-AP in this patient. The novel insights generated from this study not only improve our understanding of the complex etiology of HTG, but also have important implications for variant interpretation as well as disease prevention. For example, the study of the novel *LPL* p.Ile252Met variant clearly demonstrates that rare variants can confer a small or modest effect in the same way as common variants. Moreover, whereas the evaluation of evolutionary conservation and *in silico* pathogenicity prediction are both helpful in assessing the pathogenic relevance of missense variants, the precise underlying biological mechanisms can only be addressed by *in vitro* functional analysis. Finally, subjects found to carry any genetic risk factors for HTG should be well advised to be abstemious with regard to their alcohol consumption.

## Data Availability Statement

The original contributions presented in the study are included in the article/supplementary material, further inquiries can be directed to the corresponding author/s.

## Ethics Statement

The studies involving human participants were reviewed and approved by the Ethics Committee of Jinling Hospital. The patients/participants provided their written informed consent to participate in this study. Written informed consent was obtained from the individual(s) for the publication of any potentially identifiable images or data included in this article.

## Author Contributions

QY: conceptualization, methodology, project administration, and writing-original draft. NP: data curation, investigation, methodology, and writing-original draft. X-YL: investigation, methodology, and software. X-LS and W-WC: validation and visualization. G-FZ: software and visualization. Y-PH: investigation and software. JZ: data curation and funding acquisition. F-XC: project administration and validation. B-QL: project administration and resources. Z-HT: formal analysis and funding acquisition. CF: supervision and writing – review and editing. DC: formal analysis and writing – review and editing. J-MC: conceptualization, formal analysis, supervision, writing-original draft, and writing – review and editing. W-QL: funding acquisition, project administration, and resources. All authors contributed to revision of the manuscript and approved the final manuscript.

## Conflict of Interest

The authors declare that the research was conducted in the absence of any commercial or financial relationships that could be construed as a potential conflict of interest.

## Footnotes

1 https://gnomad.broadinstitute.org/

2 http://www.hgmd.cf.ac.uk/ac/search.html

3 https://www.ncbi.nlm.nih.gov/clinvar/

4 https://databases.lovd.nl/shared/genes/LPL

5 https://pubmed.ncbi.nlm.nih.gov/
